# Proliferation, Characterization and Differentiation Potency of Adipose Tissue-Derived Mesenchymal Stem Cells (AT-MSCs) Cultured in Fresh Frozen and non-Fresh Frozen Plasma

**DOI:** 10.22088/IJMCM.BUMS.8.4.283

**Published:** 2019

**Authors:** Wahyu Widowati, Rachmawati Noverina, Wireni Ayuningtyas, Dedy Kurniawan, Hanna Sari Widya Kusuma, Seila Arumwardana, Dwi Surya Artie, Ika Adhani Sholihah, Rr. Anisa Siwianti Handayani, Dian Ratih Laksmitawati, Ratih Rinendyaputri, Rilianawati Rilianawati, Ahmad Faried

**Affiliations:** 1 *Medical Research Center, Faculty of Medicine, Maranatha Christian University, Bandung, West Java, Indonesia.*; 2 *Animal and Stem Cells Laboratory, PT Bio Farma (Persero), Bandung 40161, West Java, Indonesia.*; 3 *Doctoral Program, Faculty of Medicine, Universitas Padjadjaran, Bandung, West Java, Indonesia.*; 4 *Magister Program, Faculty of Medicine, Universitas Padjadjaran, Bandung, West Java, Indonesia.*; 5 *Biomolecular and Biomedical Research Center, Aretha Medika Utama, Bandung, West Java, Indonesia.*; 6 *Faculty of Pharmacy, Pancasila University, Jakarta, Indonesia.*; 7 *National Institute of Health Research and Development, Ministry of Health, Jakarta, Indonesia.*; 8 *Agency for the Assesment and Application of Technology, Ministry of Research and Technology, Serpong, Indonesia.*; 9 *Department of Neurosurgery and Stem Cell Working Group, Faculty of Medicine, Universitas Padjadjaran-Dr. Hasan Sadikin Hospital, Bandung, West Java, Indonesia.*

**Keywords:** Adipose tissue-MSCs, multilineage differentiation, population doubling time, proliferation, surface marker

## Abstract

Mesenchymal stem cells (MSCs) have unique properties, including high proliferation rates, self-renewal, and multilineage differentiation ability. Their characteristics are affected by increasing age and microenvironment. This research is aimed to determine the proliferation, characteristics and differentiation capacity of adipose tissue-derived (AT)-MSCs at many passages with different media. The cell proliferation capacity was assayed using trypan blue. MSCs characterization (CD90, CD44, CD105, CD73, CD11b, CD19, CD34, CD45, and HLA-DR) was performed by flow cytometry, and cell differentiation was determined by specific stainings. Population doubling time (PDT) of AT-MSCs treated with fresh frozen plasma (FFP) and non-FFP increased in the late passage (P) (P15 FFP was 22.67 ± 7.01 days and non-FFP was 19.65 ± 2.27 days). Cumulative cell number was significantly different between FFP and non-FFP at P5, 10, 15. AT-MSCs at P4-15 were positive for CD90, CD44, CD105, and CD73, and negative for CD11b, CD19, CD34, CD45, and HLA-DR surface markers. AT-MSCs at P5, 10, 15 had potential toward adipogenic, chondrogenic, and osteogenic differentiation. Therefore, PDT was affected by increased age but no difference was observed in morphology, surface markers and differentiation capacity among passages. Cumulative cell number in FFP was higher in comparison with non-FFP in P5, 10, 15. Our data suggest that FFP may replace FBS for culturing MSCs.

Mesenchymal stem cells (MSCs) are adult stem cells that have differentiation and self-renewal capacity. Moreover, MSCs are able to give rise to mesodermal and non-mesodermal derived tissues, and participate to organ homeostasis, wound healing, and successful aging. MSCs have the ability to differentiate into osteoblasts, chondrocytes, and adipocytes (1, 2, 3).

According to international society for cell therapy (ISCT), MSCs have many criteria: they adhere to flask in culture condition; they express surface markers, presenting positive lineage cluster of differentiation CD73, CD90, and CD105, and negative lineage CD34, CD45, HLA-DR, CD14 or CD11b, CD79a or CD19 that can be analyzed by fluorescence activated cell sorting (FACS); they may differentiate into osteoblasts, adipocytes, and chondroblasts (2, 3, 4, 5, 6). These criterias were standardized for human MSCs but may be different for other species. MSCs can be isolated from dental pulp, amniotic, membrane, amniotic fluid, chori-onic membrane, chorionic villi, decidua, placenta (7, 8, 9), bone marrow, synovial tissue, lung tissue, umbilical cord blood, peripheral blood, and adipose tissue (AT) (10, 11).

The waste of liposuction surgery is the best source of MSCs because AT from this waste contains abundant AT-MSCs, with low side effects for the donor. AT-MSCs has a fibroblast-like morphology, and has the potential to differentiate into chondrocyte, adipocyte, and osteocyte (12).

AT had been isolated and characterized since 1970. It had been obtained from bovine, dog, goat, horse, rabbit, rat, mouse, pig, and human. Difference in liposuction procedures, isolation techniques, culture conditions, age, and body mass index impact the AT-MSCs isolation yield. Passaging of stem cells can cause a significant difference in capacity of differentiation and proliferation (1,13-16). The clinical application of MSCs requires extensive expansion, maintenance of their differential potentials, and prior MSCs characterization *in vitro* (17). MSCs have a limited life span, undergo senescence on long-term culture *in vitro*, and lose the differentiation potentials and character of stem cells with increasing time in culture (17). For *in vitro* expansion, MSCs need to be able to maintain their character as well as their differentiation and self-renewal capacities (17). The use of platelet rich plasma (PRP) supplement has been described, and applied extensively to increase the expansion of MSCs (6), but it showed no influence on cells' character and differentiation capacities. The objective of this research was to evaluate the effect of different media namely fresh frozen plasma (FFP) and non-FFP at various passages on cell proliferation, morphology, surface marker characteristics, and differentiation capa-cities.

## Materials and methods

**MSCs isolation from adipose tissue**

AT resulting from liposuction was put into schott bottle (250 or 500 ml) fulfilled with transport medium Minimal Essential Media with alpha modifications (MEM-α) 80% (A1049001, Gibco, USA), 1% antibiotic and antimycotic (15240062, Gibco, USA) and FFP (Indonesian Red Cross, Bandung, Indonesia) in ice bag. Sample collection has been approved by the patient by fulfilling the inform consent. All procedures has been approved by the Institutional Ethics Committee of Padja-djaran University Bandung, West Java, Indonesia (No. 1062/UN6.C1.3.2/KEP/PN/2016). After that, the fats were filtered by cell strainer 100 µm (93100, SPL, Korea) and washed with phosphate buffered saline (PBS) (14200075, Gibco, USA), then transferred into 15 ml tube (50015, SPL, Korea). Collagenase type I (30 ml) 0.075% (17100017, Gibco, USA) was added into the tube and centrifuged (MPW-260 R) at 1200 rpm, 10 min at room temperature. Then, the cell pellet was put into flask with complete medium consisting of 80% MEM-α, 20% FFP, 1% antibiotic and antimicotic, and 1% heparin (IH2983, Inviclot, Indonesia) (18).

**Passaging and cell proliferation** **analysis**

Passaging MSCs with FFP treatment were cultured 3-5 days in FFP medium, with the medium being replaced every 2 days. For non-FFP, cells were given a complete medium consisting of 80% MEM-α, 1% antibiotic and antimicotic, and 1% heparin but at the last day the cells were treated with a medium without FFP for 24 h to make the cells starve.

Cells were counted and passaged at 80% confluence. Briefly, cultured cells were detached by trypsin (25200072, Gibco, USA), then incubated for 1-3 min at 37 ^o^C in complete medium consisting of 80% MEM-α, 20% FFP, 1% antibiotic and antimicotic, and 1% heparin that were added to stop trypsin, and centrifuged at 1600 rpm for 5 min at room temperature. The pellet of cells was resuspended with trypan blue solution (25200072, Sigma Aldrich, USA) and diluted 1:1. Then, cells were counted with a hemocytometer (Neubauer, 17849). Population Doubling (PD) was counted at every passage with the formula:

$\mathrm{PD}=\left[\log_{10}\left(\mathrm{NH}\right)-\log_{10}\left(\mathrm{NI}\right)\right]/\log_{10}$

where NI is the inoculum cell number and NH is the cell harvest number. Then, to determine cumulative PD data, the PD at the previous passage was added. 

The PD time (PDT) was determined by the formula(4).

PDT = t (time)/PD (days)

**Characterization of MSCs**

MSCs from P3-P14 cultured at density of 2 x 10^6^ cells in T-flask 25 cm^3 ^(CLS430639, Corning, USA) that reached 80% confluence were harvested (P4-P15) for markers analysis using flowcytometry (Macsquant, Analyzer 10, Miltenyi Biotec, Germany). The cells were stained with specific antibodies (CD90 FIT C, CD105 PerCP-Cy5, CD73 APC, CD34 PE, CD116 PE, CD19 PE, CD45 PE, HLA-DR PE, and CD44 PE) according to manufacturers’ protocol (BD stem flow^TM^kit, 562245, USA). The experiments and measurement of surface markers were performed in triplicate (4).

**AT-MSCs differentiation**

For osteogenic differentiation, AT-MSCs were seeded at density 5 × 10^3^ cells in 24 well plate (72296-18, Nunc, USA) using StemPro Osteogenesis Differentiation Kit (A10072-01, Gibco, USA) for 3 weeks. Calcium deposits were visualized using Alizarin red S (A5533, Sigma Aldrich, USA). For chondrogenic differentiation, AT-MSCs were seeded at density 5 × 10^3^ cells in 4 well plate (176740, Nunc, USA) using StemPro Chondrogenesis Differentia-tion Kit (A10071-01, Gibco, USA) for 3 weeks. The Glycoprotein secreted by chondrocytes were visualized using alcian blue (A5268, Sigma Aldrich, USA). Adipogenic differentiation of AT-MSCs was done using StemPro Adipogenesis Differentiation Kit (A10070-01, Gibco, USA) for 3 weeks. Oil  red O (00625, Sigma Aldrich, USA) was used to conﬁrm lipid droplets (2, 3).

**Statistical analysis**

Statistical analysis was conducted using SPSS software (version 20.0). Data were presented as mean ± standard deviation. Significant differences among treatments were determined using the one-way analysis of variance (ANOVA) and P <0.05 was considered as statistically significant, along with Tukey post hoc test.

## Results

**Effect of passaging on cell morphology and duplication time**

**Fig. 1.**
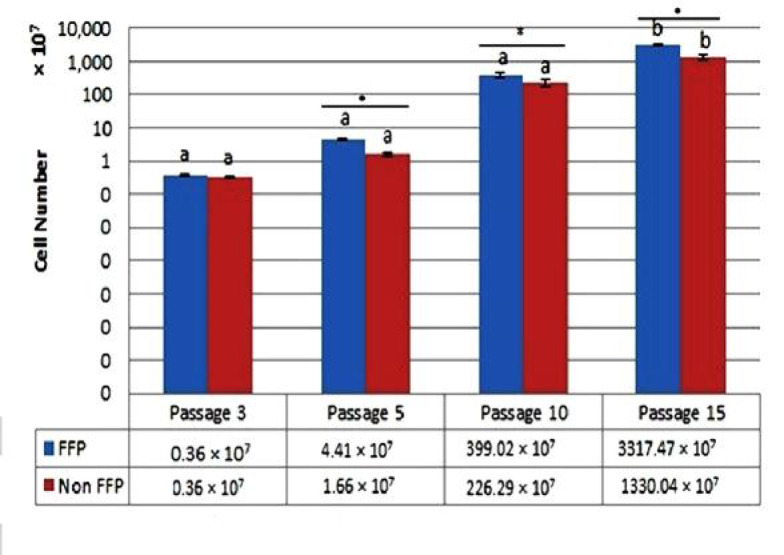
**Cumulative cell number after FFP and non-FFP treatment at P3, P5, P10 and P15. **Different superscripts of small letters (a,b) in the same color charts (blue color for FFP, red color for non-FFP treatment) were significantly different at P < 0.05 (Tukey post hoc test), each column represents the mean ± standard error of three independent experiments (*: P < 0.05). FFP: fresh frozen plasma; Non FFP: non-fresh frozen plasma

Figure 1 shows the cumulative cell number of AT-MSCs. The cell proliferation and cell number are affected by passage and microenvironment. The number of cells in the early passage (P3) was the lowest both in FFP and non-FFP. Higher passages increased the cumulative cells number.

**Table 1:** PDT and PD rate of AT-MSCs from Passage 3-15

**Passage**	**PDT (days)**		**PD rate**	
	**FFP**	**Non-FFP**	**FFP**	**Non-FFP**
**P3**	3.36 ± 0.32^a^	3.81 ± 0.41^a^	0.90 ± 0.08^cd^	0.79 ± 0.08^bcd^
**P4**	1.77 ± 0.11^a^*	3.73 ± 0.43^a^*	1.70 ± 0.10^g^*	0.81 ± 0.10^cd^*
**P5**	1.61 ± 0.03^a^*	2.10 ± 0.21^a^*	1.86 ± 0.04^fg^*	1.44 ± 0.15^fg^*
**P6**	2.47 ± 0.03^a^	2.35 ± 0.19^a^	1.62 ± 0.02^fg^	1.78 ± 0.02^g^
**P7**	3.00 ± 0.48^a^	2.43 ± 0.21^a^	1.36 ± 0.21^ef^	1.65 ± 0.14^g^
**P8**	3.82 ± 0.48^a^	3.52 ± 0.22^a^	1.32 ± 0.18^ef^	1.42 ± 0.09^fg^
**P9**	4.42 ± 0.32^ab^	3.97 ± 0.68^a^	1.14 ± 0.09^de^	1.41 ± 0.02^ef^
**P10**	6.77 ± 1.13^ab^	7.02 ± 0.42^b^	1.06 ± 0.19^de^	1.00 ± 0.06^de^
**P11**	8.09 ± 0.51^ab^	9.35 ± 1.42^bc^	0.87 ± 0.05^cd^	0.84 ± 0.12^bcd^
**P12**	9.21 ± 1.99^abc^	12.20 ±1.02^cd^	0.78 ± 0.15^bcd^	0.58 ± 0.05^abc^
**P13**	11.91 ± 3.24^bc^	14.63 ± 1.92^d^	0.78 ± 0.20^abc^	0.48 ± 0.07^ab^
**P14**	15.95 ± 5.01^cd^	18.08 ± 0.72^e^	0.46 ± 0.12^ab^	0.39 ± 0.02^a^
**P15**	22.67 ± 7.01^d^	19.65 ± 2.27^e^	0.33 ± 0.10^a^	0.36 ± 0.04^a^

Data are presented as mean ± standard deviation. * indicates significant difference between the means of PDT and PD rate for FFP and non-FFP treatment in various passages (P < 0.05 based on T-test). Different superscript small letter (a,b,c,d,e,f,g) in same column are significant difference among the means of groups (among various passage both in PDT and PD rate of FFP and non-FFP) at P < 0.05 based on Tukey post hoc test. FFP: fresh frozen plasma; Non-FFP = non-fresh frozen plasma

The PDT and PD rates are represented in Table 1. PDT and PD rates were used to evaluate the MSCs cell proliferation capacities; PDT is the time for cells to divide themselves. The faster the cell proliferation, the lower the PDT value is (Table1). PDT assay of AT-MSCs (FFP and non-FFP), showed that higher passages increased PDT value, and the ability of cell proliferation depended on the number of passage. The lowest PDT or faster cell proliferation in FFP was P3 to P8 (1.61-3.82 days) and P3 to P8 (2.10-3.97 days) for non-FFP. The lowest PD rate in FFP was P15 (0.33) and P14 to P15 (0.36-0.39) for non-FFP. Based on t-test, there were significant differences in P4, P5 for both PDT and PD rates between FFP and non-FFP.

**Fig. 2:**
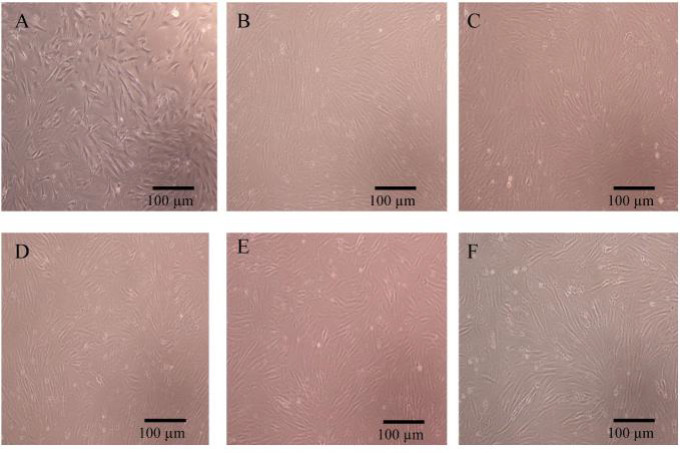
**Morphology of AT-MSCs from one representative donor at early passage (P5),** **moderate passage (P10), and late passage (P15).** A: P5 FFP; B: P10 FFP; C: P15 FFP; D: P5 non-FFP; E: P10 non-FFP; F: P15 non-FFP. Magnification: 100×. FFP: fresh frozen plasma; Non FFP: non-fresh frozen plasma

Cell morphology is illustrated in Figure 2. The morphology of P5, P10, P15 of AT-MSCs were not different. These figures exhibited plastic adherent properties, fibroblast-like morphology, and spindle-shaped cells. Cell morphology of the early (P5), moderate (P10) and late passages (P15) in both FFP and non-FFP treatment did not show any difference.

**Table 2:** Percentage of lineage-positive and negative surface markers of AT-MSCs

**Passage**	**CD90 (%)**	**CD44 (%)**	**CD105 (%)**	**CD73 (%)**	**Lineage- negative (%)**
**4**	98.58 ± 2.01	99.53 ± 0.03	99.63 ± 0.05	90.54 ± 0.51	3.96 ± 0.00
**5**	99.42 ±0.05	99.42 ± 0.03	99.55 ± 0.03	96.77 ± 0.17	0.23 ± 0.02
**6**	99.18 ± 0.97	99.14 ± 0.99	99.30 ± 0.99	98.54 ± 0.98	0.39 ± 0.06
**7**	99.70 ± 0.06	99.70 ± 0.06	99.76 ± 0.06	98.95 ± 0.12	0.20 ± 0.06
**8**	99.66 ± 0.04	99.71 ± 0.03	99.75 ± 0.06	98.35 ± 0.37	0.11 ± 0.05
**9**	99.33 ± 0.07	99.39 ± 0.10	99.45 ± 0.08	98.62 ± 0.11	0.21 ± 0.05
**10**	98.75 ± 0.13	98.76 ± 0.21	98.85 ± 0.22	97.72 ± 0.34	0.70 ± 0.05
**13**	97.60 ± 0.06	97.58 ± 0.14	97.39 ± 0.05	95.21 ± 0.11	2.18 ± 1.08
**14**	98.99 ± 0.09	98.93 ± 0.13	99.04 ± 0.18	98.73 ± 0.21	0.42 ± 0.09
**15**	99.72 ± 0.03	99.75 ± 0.05	99.78 ± 0.04	99.30 ± 0.11	0.76 ± 0.58

Data are expressed as mean ± standard deviation from 3 replications; CD90, CD44, CD105, and CD73 are lineage-positive markers; CD11b, CD19, CD34, CD45, and HLA-DR are lineage-negative markers

This result indicates that passaging only affects the increase of the cell number, but not the cell morphology.

**Effect of FFP and non-FFP on cell surface mar-kers**

**Fig. 3:**
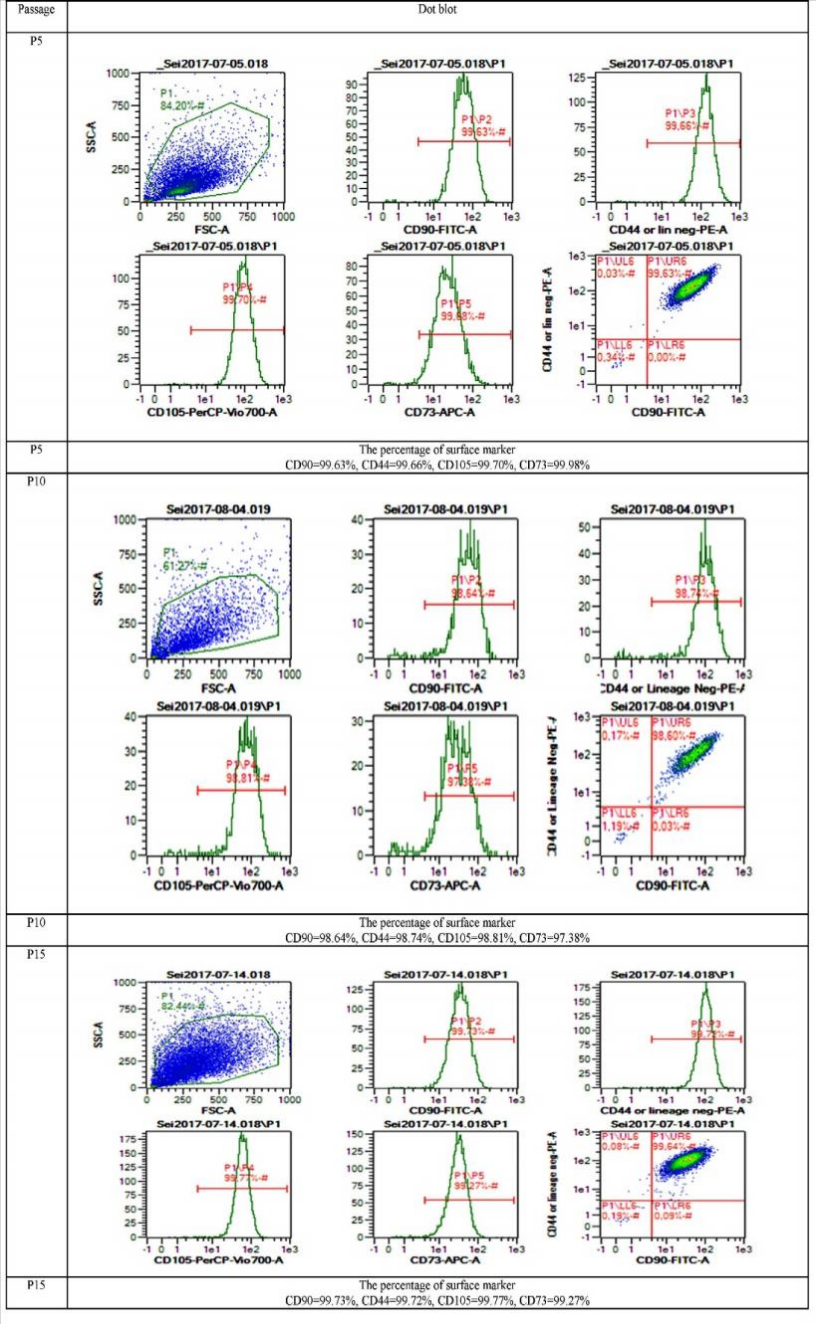
**Dot blot representative of AT-MSCs surface markers CD105, CD90, CD44, CD105, CD73.** A: passage 5 (P5); B: passage 10 (P10); C: passage 15 (P15) in FFP supplementation

MSCs should have lineage marker positive CD44, CD73, CD90, and CD105 and lineage marker negative CD11b, CD19, CD34, CD45, and HLA-DR. The surface markers of AT-MSCs were detected using fluorescein staining PE (propyl etidium) and FITC (fluorescein isothiocyanate) (Table 2, Figure 3). FFP supplement proved that it was effective to increase proliferation and did not affect the characteristics of MSCs. Table 2 and figure 3 represent the AT-MSCs characterizations. AT-MSCs at P4 to 15 showed the MSCs’ characteristics. Moreover, Table 2 showed that P15 FFP still have MSCs characteristics as the P15 population was 99.72% CD90, 99.78% CD105, 99.30% CD73, and 99.75% CD44 positive, and 0.76% lineage-negative for CD11b, CD19, CD34, CD45, and HLA-DR.

**Fig. 4.**
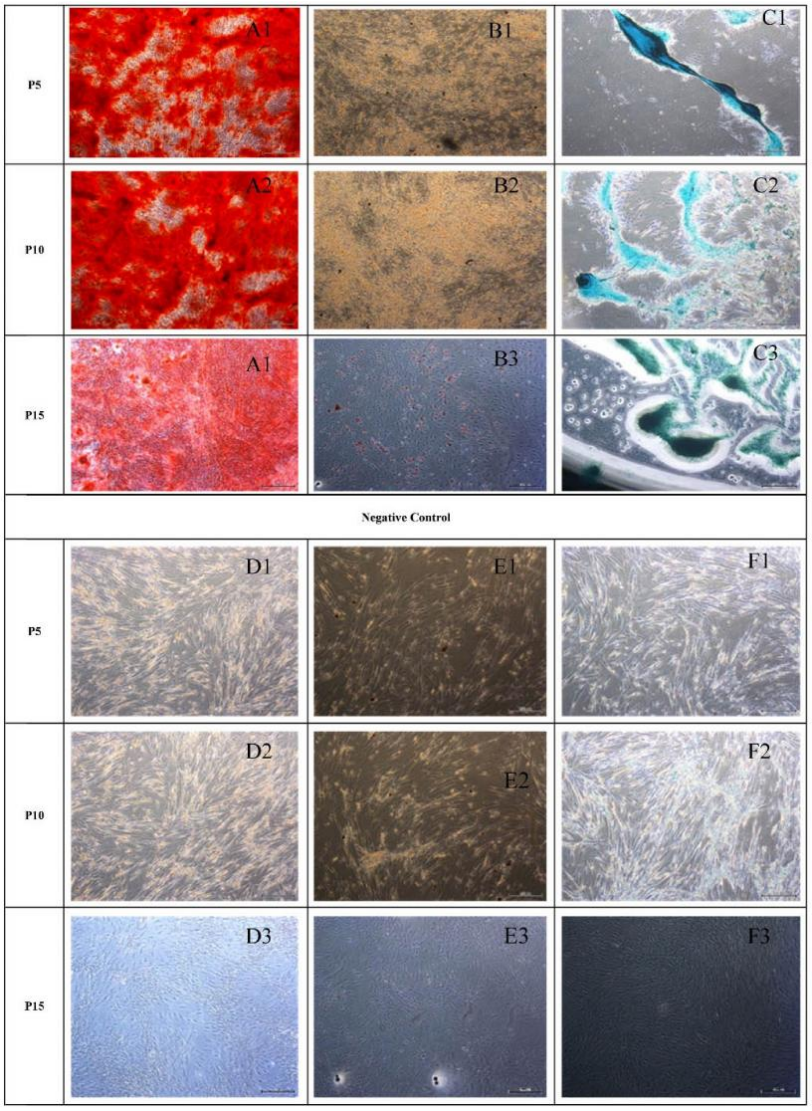
**Morphological appearance of osteogenic, adipogenic and chondrogenic differentiation of AT-MSCs.** Result of AT-MSCs differentiation (after staining) was indicated by A, B and C. Undifferentiated AT-MSCs (negative control) were indicated by D, E, and F. A: osteogenic (A1: P5; A2: P10; A3: P15); B: adipogenic (B1: P5; B2: P10; B3: P15); C: chondrogenic (C1: P5; C2: P10; C3: P15); D: control of osteogenic ; E: control of adipogenic; F: control of chondrogenic

**Effect of FFP and non-FFP on AT-MSCs** **diffe-rentiation **

AT-MSCs at early (P5), moderate (P10), and late (P15) passage differentiated into osteocytes, chondrocytes, and adipocytes (Figure 4). The adipogenic, osteogenic, and chondrogenic differentiation abilities may be enhanced by specific differentiation media. Osteogenic differe-ntiation was confirmed by staining with alizarin red S after 3 weeks. The calcium deposits were visualized with alizarin red S (Figure 4A). Adipogenic differentiation was revealed after 3 weeks by staining with oil red-O. Oil red-O was used to visualize neutral lipid accumulation (Figure 4B). Chondrogenic differentiation was visualized by alcian blue staining (Figure 4C).

## Discussion

In a previous study, PD of umbilical cor blood (UCB)-MSCs, AT-MSCs, bone marrow (BM)-MSCs and wharton jelly (WJ)-MSCs were measured for every passage. The longest period and the best development capacity were shown by UCB-MSCs. The proliferation of UCB-MSCs declined at P14-16. While AT-MSCs showed the shortest culture time and lowest growth capacity, they were stopped at P11-12 (19). In comparison with our results, the proliferation of AT-MSCs were stable and high at P1 to 8 for FFP and P1 to 9 for non-FFP medium, then it decreased at P 10 to 15. 

In the present study, the cell proliferation and cell number were affected by passage and microenvironment. A significant difference was observed between FFP and non-FFP in P4, P5 for both PDT and PD rate (Table 1). This resultt was in line with the previous study where both AT-MSCs and BM-MSCs showed good enough proliferation rates in P2, 5, and 8, especially at P2 and 8, but proliferation decreased gradually at older passages (20). This result was supported by a previous research revealing that WJ-MSCs treated either in normoxic or hypoxic (O_2_ 2.5%; O_2_ 5%) conditions showed lower PDT at early passages (P1-P5), and had higher PDT at moderate passages (P6-P8) (4). FFP medium showed lower PDT in comparison with non-FFP medium on P4-P5. FFP is a blood product made from the liquid portion of whole blood, and is used as part of plasma exchange. It is a complex mixture of water, proteins, carbohydrates, fats, and vitamins (21), and has characters similar to PRP, PRP contains various growth factors such as platelet-derived growth factor-AB (PDGF-AB) 47,096.63 pg/mg protein, transforming growth factor-β1 (TGF-β1) 74,817.76 pg/mg protein, insulin-like growth factor-1 (IGF-1) 287.89 pg/mg protein, and vascular endothelial growth factor (VEGF) 150.93 pg/mg protein (6). The WJ-MSCs cultured in medium supplemented with human platelet from donors with blood group O and AB (hPL-ABO) had lower PDT in comparison with FBS medium in P 1 to 8 (6). Growth factors released by platelets in hPL-ABO are effective to stimulate proliferation of WJ-MSCs (6). Different serua has been tested to increase proliferation of AT-MSCs such as autologous non-activated PRP (nPRP) and thrombin-activated PRP (tPRP). Atashi et al. (2014) suggested that 20% nPRP improved cell proliferation. This concentration increased the proliferation of AT-MSCs by 13.9 times without changing the AT-MSCs phenotype, differentiation capacity, and chromosome status (22). Furthermore, human AB serum (AB-HS) has significant effect to AT-MSCs proliferation in comparison withs fetal calf serum (FCS) (23). Antonius et al. (2015) suggested that high and stable cell proliferation rate of WJ-MSCs in the hPL-ABO-supplemented culture medium resulted in higher cumulative cell number in comparison with those cultured in FBS-supplemented medium (6). In another study, there was similarity in doubling times and cumulative population doublings in MSCs cultured in medium supplemented with 10% AB-HS in comparison with 10% FBS-supplemented culture (24). Human blood-derived components such as human serum (autologous or pooled allogeneic), platelet products, and umbilical cord blood serum seem to be the most efficient and safer, and can be considered a promising FBS alternative (25, 26). In this study, FFP was used as supplement medium. FBS in MSCs may pose an infection risk, which induce immunological reactions in the host for therapy application (27). Thus, regarding a significant safety concern when administering MSCs cultured in FBS, many researchers try to change the FBS use in MSCs culture for therapy application. It has also been reported that patients who have received cell transplantation with MSCs expanded in FBS exhibited antibodies against bovine antigens (28).

The present data showed that the proliferation of AT-MSCs was higher using FFP than non-FFP. At P4, the cumulative cell number for FFP treatment was higher than non-FFP treament (Table 1). The human AB serum and tPRP are alternatives to FCS for AT-MSCs (24). In another study, autologous serum increased the proliferation rates (29). PRP can produce chemokines; cytokines and growth factors can promote the recruitment, adhesion, and proliferation of adult stem cells (30). To eliminate the use of animal products in human AT-derived stem cells (ADSCs) cultures, PRP can substitute the FBS as PRP contains a wide range of proteins, growth factors, and enzymes supporting attachment, growth, and proliferation of cells (31, 32).

Based on data presented in Figure 1, the passaging only affects cells' doubling time, but not cells morphology (Figure 2). Ren et al. (2016) compared human UC-MSCs, dental pulp (DP)-MSCs and menstrual blood (MB)-MSCs as source for cell therapy, and found that along cell passaging, flattened cells adopted a polygonal shape. DP-MSCs seemed to keep a good state in fibroblast-like morphology at each passage (33). 

In the present study AT-MSCs at P4 to 15 were positive for the hMSCs markers CD90, CD44, CD105, and CD73; (more than 95%) and negative for CD11b, CD19, CD34, CD45, and HLA-DR (less than 2%). This data was validated with previous researches showing that WJ-MSCs highly expressed CD90, CD44, CD105, and CD73 and had low expression of CD34, CD45, CD14, CD19, and HLAII at both early (P4) and moderate passage (P8) (2, 4, 6). Also, it was shown that MSCs from various sources, including AT-MSCs were positive for CD73, CD90, and CD105 and negative for CD11b or CD14, CD19, CD34, CD45, and HLA-DR in their cell surface (34).

Pluripotency was confirmed by the ability of WJ-MSCs cells to differentiate into osteocytes, chondrocytes, and adipocytes (2, 3). In this study AT-MSCs at early (P5), moderate (P10) and late passage (P15) differentiated into osteocytes, chondrocytes, and adipocytes (Figure 4). These data were validated with a previous finding that MSCs could be expanded to 10 or 11 passages (35). The adipogenic or osteogenic differentiation are upregulated by *OCT4*/*SOX2* overexpression. OCT4 and SOX2 transcription factors as adipogenic and osteogenic markers, are naturally expressed in MSCs for pluripotency and self-renewal at low levels in early passages (36). Cells began to show a round shape and most of them contained cytoplasmatic vacuoles, intracellular accumulated lipids, and small oil droplets in the cytoplasm that were positive with oil red O staining (2, 6). Osteogenic induction medium can induce the production of mineralized matrix, and cells displaying bone-like nodular aggregates of matrix mineralization can be detected by alizarin red S staining (2, 6) while chondrogenic induction medium forms an acidic proteoglycans component which can be detected by alcian blue staining (2, 6, 37, 38).

In conclusion, according to the present research, PDT evaluation in two treatments (FFP and non-FFP) showed that the last passages had higher PDT in comparison with early passages and the characteristic of MSCs remained still unchanged in passages 4 to 15. AT-MSCs are capable to differentiate into osteocytes, adipocytes and chondrocytes. PDT was affected by increasing age, while there was no difference in morphology, surface markers and differentiation capacity among passages. Cumulative cell number in FFP was higher in comparison with non-FFP. FFP may be a promising substance to replace FBS for obtaining MSCs free FBS. 
